# Retrieval Research in Hip and Knee Arthroplasty

**DOI:** 10.1155/2016/3480840

**Published:** 2016-10-16

**Authors:** Thomas M. Grupp, Sandra Utzschneider, Steven M. Kurtz

**Affiliations:** ^1^Aesculap AG Research and Development, 78532 Tuttlingen, Germany; ^2^Department of Orthopaedic Surgery, Physical Medicine and Rehabilitation, Ludwig Maximilians University Munich, Campus Grosshadern, 81377 Munich, Germany; ^3^Implant Research Center, Drexel University and Exponent, Inc., Philadelphia, PA, USA

During the last two decades, improvements in hip and knee designs, bearing materials, sterilisation techniques, oxidation stabilisation, and articulating surface treatments have led to superior performance of total hip and knee arthroplasties by reducing the prevalence of wear, delamination, and structural material fatigue. These advances are expected to show substantial benefits in decreasing wear, osteolysis, and improved joint function in the coming decades.

In contrast to that new implant designs, articulating bearings, implant modularities, kinematic concepts, and surgical treatments came up, but not all of them were as beneficial in regard to their expected service in vivo in the 2nd and 3rd decade, patient satisfaction, and clinical outcomes.

As total hip and knee arthroplasty today is being increasingly performed on younger, heavier, and more active patients, it appears desirable to further improve implant designs, modular connections, bearing materials, and implant fixation methods to allow for a higher degree of function, patient satisfaction, and long-term survivorship. Dedicated retrieval research programs are a main source to gain more knowledge about the complex surgeon-implant-patient interactions and a deeper understanding on material degradation and potential adverse side effects in vivo to create sustainable arthroplasty technologies for the future.

This special issue presents 7 articles with original research papers and clinical study findings showing different dimensions of implant revision and retrieval research in hip and knee arthroplasty.

In retrieval analysis on polyethylene inlays of an open acetabular screw ring cup design showing a high clinical failure rate, U. Mueller et al. examine the specific failure mechanism and concluded that ongoing creep deformation due to insufficient cup support leads to substantial backside wear, collar fatigue, and decreased clearance.

To understand the wear process of polyethylene inlays from acetabular cup systems with a cone press-fit locking mechanism A. L. Puente Reyna et al. evaluate the backside wear characteristics in a direct comparison of wear simulator testing and retrievals. Similar wear scores for in vitro tested specimen and retrievals (in situ) were found with insertion and removal of the inlay as main source of backside wear.

Using an artificial neural network approach the research paper of D. A. Orozco Villasenor and M. A. Wimmer describes a new method to identify wear scar similarities and discrepancies between retrieved and simulator tested polyethylene gliding surfaces and suggested that current ISO knee testing protocols were not fully representative for in vivo behaviour.

J. A. Eckert et al. performed a retrieval analysis about tribocorrosion in modular shoulder arthroplasty taper junctions using a modified Goldberg score and found the prevalence of fretting and corrosion with titanium stems being more susceptible to tribocorrosion damage than cobalt-chromium stems.

A. C. Paulus et al. have undergone a histopathological analysis of wear particle effects on the synovial tissue achieved during knee revision surgery of a rotating hinge knee design with CFR-PEEK implant components and found differences to previous animal studies. Furthermore they describe specific differences between the agglomeration behaviour of polyethylene and PEEK in human synovial tissue.

Periprosthetic infection is a remaining major complication in orthopaedic oncology limb salvage surgery procedures of pathologic fractures due to bone metastases. By F. Donati et al., a retrospective clinical study on bone tumor patients (follow-up of 46.5 months) treated with a silver coated or uncoated proximal femur replacement hip hemiarthroplasty was presented, demonstrating good antimicrobial activity with a lower rate of early infection in the silver coated group and low toxicity.

As 10 to 20 percent of TKA patients are dissatisfied with their clinical outcome this is a main reason for early knee revision. A. Giurea et al. studied the impact of personality traits in a cohort of computer navigated TKA patients based on 12 personality traits tested by the Freiburg Personality Inventory (FPI-R). Within this TKA cohort with optimal alignment, 16% of patients were dissatisfied and the FPI-R showed a substantial influence of the personality traits life satisfaction, performance orientation, somatic distress, and emotional stability on clinical outcome and patient satisfaction after TKA.

Finally the guest editors would like to thank all authors for contributing their excellent work to this special issue and all the reviewers for their thoughts and suggestions on the manuscripts.

*Thomas M. Grupp* *Sandra Utzschneider* *Steven M. Kurtz*

